# Activation of protease activated receptor 1 increases the excitability of the dentate granule neurons of hippocampus

**DOI:** 10.1186/1756-6606-4-32

**Published:** 2011-08-10

**Authors:** Kyung-Seok Han, Guido Mannaioni, Cecily E Hamill, Jaekwang Lee, Candice E Junge, C Justin Lee, Stephen F Traynelis

**Affiliations:** 1 Department of Pharmacology, Emory University School of Medicine, Atlanta, GA, USA; 2 Center for Neural Science and Functional Connectomics, Korea Institute of Science and Technology (KIST), Seoul, Korea; 3 Neuroscience Program, University of Science and Technology, Daejeon, Korea

## Abstract

Protease activated receptor-1 (PAR1) is expressed in multiple cell types in the CNS, with the most prominent expression in glial cells. PAR1 activation enhances excitatory synaptic transmission secondary to the release of glutamate from astrocytes following activation of astrocytically-expressed PAR1. In addition, PAR1 activation exacerbates neuronal damage in multiple *in vivo *models of brain injury in a manner that is dependent on NMDA receptors. In the hippocampal formation, PAR1 mRNA appears to be expressed by a subset of neurons, including granule cells in the dentate gyrus. In this study we investigate the role of PAR activation in controlling neuronal excitability of dentate granule cells. We confirm that PAR1 protein is expressed in neurons of the dentate cell body layer as well as in astrocytes throughout the dentate. Activation of PAR1 receptors by the selective peptide agonist TFLLR increased the intracellular Ca^2+ ^concentration in a subset of acutely dissociated dentate neurons as well as non-neuronal cells. Bath application of TFLLR in acute hippocampal slices depolarized the dentate gyrus, including the hilar region in wild type but not in the PAR1-/- mice. PAR1 activation increased the frequency of action potential generation in a subset of dentate granule neurons; cells in which PAR1 activation triggered action potentials showed a significant depolarization. The activation of PAR1 by thrombin increased the amplitude of NMDA receptor-mediated component of EPSPs. These data suggest that activation of PAR1 during normal function or pathological conditions, such as during ischemia or hemorrhage, can increase the excitability of dentate granule cells.

## Background

Protease activated receptor 1 (PAR1) is a G-protein coupled receptor that is best known for its role in coagulation and homeostasis [1-3]. PAR-1 is activated when serine proteases such as thrombin or plasmin cleave the N-terminus at Arg41, revealing a new N-terminus that acts as a tethered ligand to activate receptor signaling [4,5]. PAR-1 signals through multiple G-proteins, including Gi, Gq, and G_12/13 _[5-7], and is highly expressed in astrocytes throughout the CNS [8-10], and differentially expressed in neuronal subpopulations in discrete regions, including granule cell layer of dentate gyrus [8,11,12]. Activation of PAR1 leads to profound changes in astrocyte function, such as the proliferation [13-15] that underlies glial scar formation in response to penetrating head wound [16]. Activation of astrocytic PAR1 also triggers the release of glutamate and subsequent potentiation of neuronal NMDA receptors secondary to depolarization-induced relief of Mg^2+ ^block [9,16,17]. In the present study, we have investigated the functional expression of PAR1 in granule cells of the dentate gyrus. We show that PAR1 activation leads to granule cell depolarization and potentiation of synaptically-activated NMDA receptor function.

## Results

### PAR1 expression in dentate granule cells

**Figure 1:**
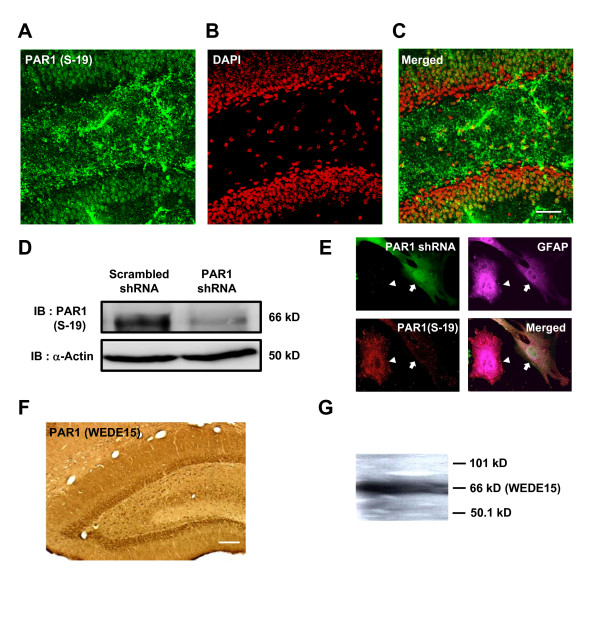
**Immunohistochemistry reveals that PAR1 receptor is expressed in dentate granule cells**. **A, B, C) **Image of dentate in a thin section from a C57Bl/6 mouse, immunostained with thrombin receptor polyclonal antibody (S-19, Santa Cruz). Red indicates DAPI staining (A) and green indicates PAR1 immunostaining (B). PAR1-immunoreactivity is clearly expressed in the dentate granule cell layer (C); scale bar: 50 μm. Data are representative of three different experiments from two different mice. **D) **Western blot of PAR1 with thrombin receptor polyclonal antibody (S-19, Santa Cruz) in cultured astrocytes transfected with scrambled shRNA or PAR1 shRNA. 66 kDa PAR1 and 50 kDa actin bands are presented. This experiment was performed three times from three different mice. **E) **Immunocytochemistry of PAR1 with thrombin receptor polyclonal antibody (S-19, Santa Cruz) in cultured astrocytes. Green indicates cultured astrocytes transfected with PAR1 shRNA. Magenta shows GFAP, a marker for all GFAP-positive astrocytes. Red represents PAR1 immunostaining. The arrow indicates a cultured astrocyte that expresses PAR1 shRNA, whereas the arrowhead indicates an astrocyte without expression of shRNA. Staining in cultured astrocyte was performed in permeabilized conditions. This experiment was performed three times from three different mice. Scale bar is 50 μm. **F) **Immunostaining with PAR1 antibody (WEDE15) in the dentate gyrus using DAB staining; scale bar is 150 μm. **G) **Western blot of PAR1 with PAR1 monoclonal antibody (WEDE15, Immunotech) in mouse whole brain homogenates. A single band corresponding to the predicted molecular weight of PAR1 (66 kDa) was observed.

Multiple studies indicate that PAR1 mRNA is expressed in both neurons and glia in a variety of brain regions [11,12]. Functional evidence exists for PAR1 expression in brain regions expressing PAR1 mRNA, such as the cortex, basal ganglia, and hippocampus [16-20]. PAR1 immunoreactivity has not previously been reported in the dentate gyrus [21], although *in situ *hybridization studies suggest PAR1 mRNA is expressed in the dentate [8,9] and injury stimulates dentate microglial proliferation that can be mimicked by PAR-1 activation [22]. To explore the potential role of PAR1 in the dentate gyrus, we evaluated the protein expression level of PAR1 using immunohistochemistry. Immunostaining with thrombin receptor polyclonal antibody (S-19; see *Methods*) showed that PAR1 was expressed in the outer layer of dentate granule cells in wild type mouse (Figure 1A~C). Immunoreactivity could also be observed in a subset of cells within the hilar region, as well as within the neuropil in the dentate molecular layer. To evaluate the specificity of thrombin receptor polyclonal antibody (S-19), we performed western blotting in cultured astrocyte transfected with scrambled shRNA or PAR1 shRNA. The efficiency of knockdown by PAR1 shRNA has been previously reported [23]. Immunoblotting showed that PAR1 shRNA reduced a single band on the western blot using S19, which we interpret to be PAR1. Immunocytochemistry with the thrombin receptor polyclonal antibody (S-19) showed that shRNA for PAR1 significantly down-regulated PAR1 immunoreactivity in cultured astrocytes (Figure 1E), suggesting that the antibody is selective for PAR1. To further investigate the expression pattern of PAR1 in the dentate gyrus, we perform immunostaining using another thrombin receptor antibody (monoclonal WEDE15) that has been previously characterized [8] (Figure 1F~G). Immunoblotting with the thrombin receptor antibody (WEDE15) shows single band of PAR1 (66 kDa) in platelets (not shown) and brain tissue (Figure 1G), suggesting the antibody selectively labels PAR1. WEDE15 staining was apparent in dentate granule cells (n = 3 separate experiments), and blocked by peptide matching the epitope (data not shown). When we checked the specificity of the antibodies, there was no staining for PAR1 if the primary or secondary antibody was omitted (data not shown). Thus, immunostaining using 2 different antibodies shows similar expression pattern of PAR1 in the dentate gyrus. These data suggest that PAR1 protein is expressed in the dentate gyrus.

### PAR1 activation in acutely dissociated dentate neurons increases intracellular [Ca^2+^]

**Figure 2:**
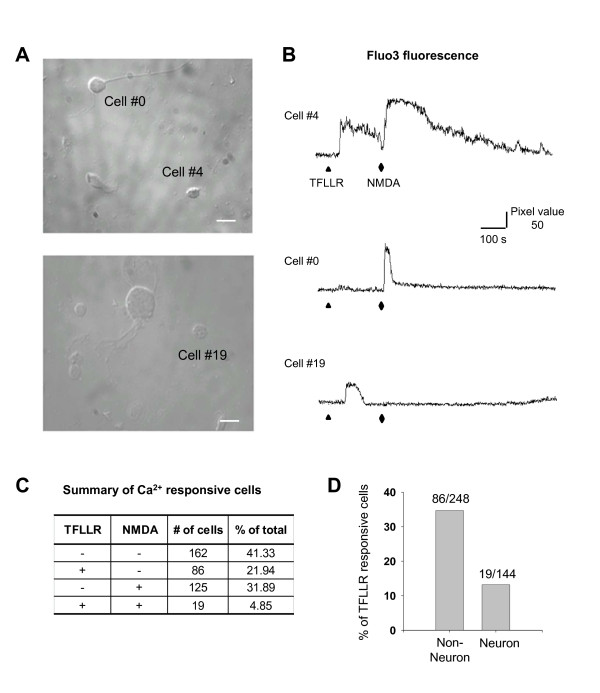
**PAR1 activation induces increase in intracellular [Ca^2+^] in a subpopulation of acutely dissociated dentate neurons, as well as non-neuronal cells**. **A) **Microphotographs of acutely dissociated cells from the dentate gyrus; scale bar is 10 μm. **B) **Ca2+ imaging traces for cell #0, #4, and #19 as shown in A. Cells were exposed to 30 μM TFLLR and 100 μM NMDA at the indicated times. Y axis indicates intensity of changes in pixel changes. **C) **Total percentages of responsive cells are expressed as percent of total cells. **D) **The response to NMDA was used to categorize the cell as neuronal or non-neuronal. The bar graph indicates the percent of TFLLR-responsive cells expressed as percent of total number of neurons or non-neurons. This experiment was performed from five different mice.

Activation of PAR1 can initiate signaling through Gα_i/o_-, Gα_q/11_-, and Gα_12/13_-coupled pathways [5,7]. To test whether PAR1 activation induces an increase in intracellular Ca^2+ ^concentration in glia and neurons from the dentate gyrus, we performed Ca^2+ ^imaging in acutely dissociated cells from the dentate that were subsequently loaded with the Ca^2+^-sensitive dye Fluo3-AM (Figure 2A). Acute dissociation ensures representative proportions of glia and neurons, and avoids changes in PAR1 expression that could artifactually occur under culture conditions. Fluo-3 fluorescence intensity was increased in over half of the individual acutely dissociated cells by the application of TFLLR or NMDA (Figure 2B). Previously, we have shown that TFLLR and thrombin did not increase intracellular Ca^2+^, and glutamate was not released by TFLLR in cultured PAR1-/- mouse astrocytes [17]. This set of results indicates a selective action of TFLLR and thrombin on PAR1. Responses to NMDA were used to distinguish between neuronal and non-neuronal cells. Four groups of cells were categorized by Ca^2+ ^responses induced by treatment of TFLLR and NMDA (Figure 2C). We found that 86 of 248 NMDA-insensitive cells responded to the PAR1 agonist TFLLR with an increase in intracellular Ca^2+^, which we interpret to be glial cells. In addition, 19 of 144 NMDA-responsive cells, which we interpret to be neurons, showed a TFLLR-induced increase in intracellular Ca^2+ ^(Figure 2D). Most of these cells had cell body diameter of less than 10 μm, consistent with the size of dentate granule cells (Figure 2A, e.g. cell #4). These results indicate that PAR1 activation leads to an increase in intracellular Ca^2+ ^in both small neurons from the dentate gyrus as well as non-neuronal cells. Furthermore, these results are consistent with a number of previous reports suggesting that thrombin stimulates Ca^2+ ^mobilization in both neurons and glia [8,10,15,16,24-27].

#### PAR1 activation depolarizes dentate gyrus in wild type but not in PAR1 -/- mice

**Figure 3:**
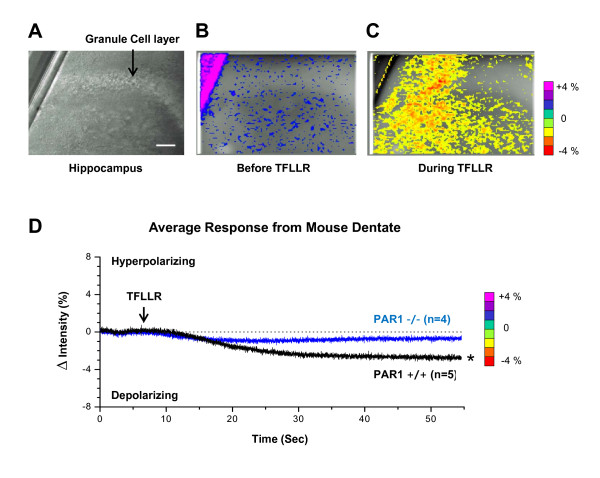
**Voltage-dye recordings show that PAR1 activation depolarizes the dentate in hippocampal slices from wild type but not in PAR1-/- mice**. **A) **Normarski image of mouse dentate within a hippocampal slice, loaded with the voltage-dye, di-4-ANEPPS and bathed in TTX (0.5 uM). Scale bar is 100 μm. **B) **Threshold-modified fluorescence voltage-dye image superimposed on a Normarski image of mouse dentate gyrus before 30 μM TFLLR was added. **C) **During application of 30 μM TFLLR, the entire dentate region becomes depolarized, represented by yellow to red colors. **D) **Average traces of wild type and PAR1-/- dentate from regions of interest over the cell body layer show that wild type dentate exhibits significantly greater depolarization, compared to the PAR1-/- dentate (p < 0.05, t-test at 55 sec). The experiment was performed from two different mice respectively.

To determine whether PAR1 activation alters neuronal function in the dentate, we performed voltage-sensitive dye imaging in the dentate gyrus of mouse hippocampal slices loaded with the voltage-sensitive dye di-4-ANEPPS and treated with 0.5 μM TTX to block action potential generation and propagation (Figure 3A~B). Bath application of the PAR1 agonist TFLLR decreased the intensity of di-4-ANEPPS fluorescence throughout the dentate gyrus, which we interpret to reflect membrane depolarization both in cell body as well as in neuronal processes (Figure 3C). By contrast, TFLLR had minimal effect on di-4-ANEPPS fluorescence in hippocampal slices from PAR1-/- mice (Figure 3D). The extent of changes di-4-ANEPPS fluorescence was significantly higher in wild-type compared to PAR1 -/- mice (p < 0.05, unpaired t-test, n = 4-5). These data are consistent with the idea that activation of PAR1 in the dentate can lead to depolarization of granule cells in the dentate gyrus.

### PAR1 activation triggers action potential generation in a subset of dentate granule cells

**Figure 4:**
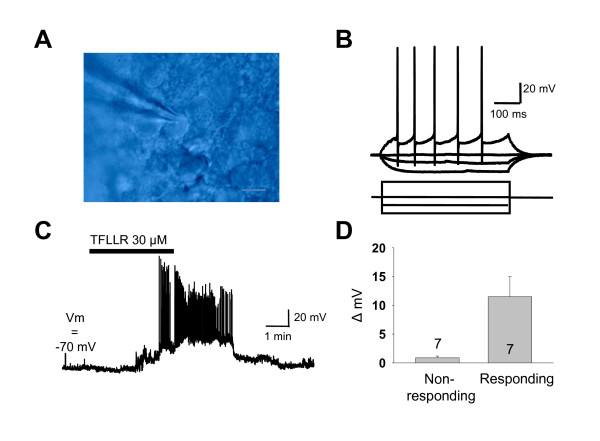
**Whole-cell recordings of dentate granule cells show that PAR1 activation causes depolarization in an individual neuron**. **A) **Image of a visually-identified dentate granule cell, patched under whole-cell configuration. Scale bar is 10 μm. **B) **The cell was injected with current as indicated to induce action potentials. Current was injected from -60 pA to 60 pA. **C) **The cell was subsequently challenged with 30 μM TFLLR, which induced a slow depolarization that drove the cell to fire action potentials repetitively. **D) **Out of 14 cells, the 7 neurons that showed spike firing activity had an average depolarization of 11.5 ± 3.5 mV (mean ± SEM), induced by TFLLR. The internal solution contained K-gluconate (see Methods).

We subsequently performed whole cell current clamp recording from dentate granule cells in hippocampal slices to investigate whether individual neuronal function was detectably influenced by PAR1 activation. Individual dentate granule neurons were visually identified using Normarski optics, recorded using patch clamp methods, and evaluated under current clamp for action potential generation in response to depolarizing current injection to verify that they were neurons (Figure 4A, B). The granule cells had a mean resting potential of -70 ± 1 mV and an input resistance of 383 ± 34 MOhm (n = 14). Resting cells did not spontaneously fire action potentials. Activation of PAR1 by application of 30 μM TFLLR induced a clear depolarization of more than +3 mV in 7 of 14 granule cells; the distribution of the change in membrane potentiation was not normally distributed. On average, there was a +8.5 mV depolarization during the application of TFLLR, which was statistically significant (Wilcoxon rank sign test, p < 0.001). Notably, there was a prominent increase in action potential generation in several of the cells showing greater than 3 mV depolarization (Figure 4C, D). The relative proportion of cells that depolarized by more than 3 mV (50%) is higher than that of acutely isolated NMDA-response cells that express functional PAR1 (13%), suggesting that PAR1-mediated granule cell depolarization may involve both intraneuronal signaling in addition to PAR1-mediated astrocytic release of glutamate. These results indicate that PAR1 can control neuronal firing and membrane potential in a subset of dentate granule neurons, thus could play a key role in controlling neuronal excitability.

### PAR1 activation increases NMDA receptor-mediated EPSPs in dentate granule cells

**Figure 5:**
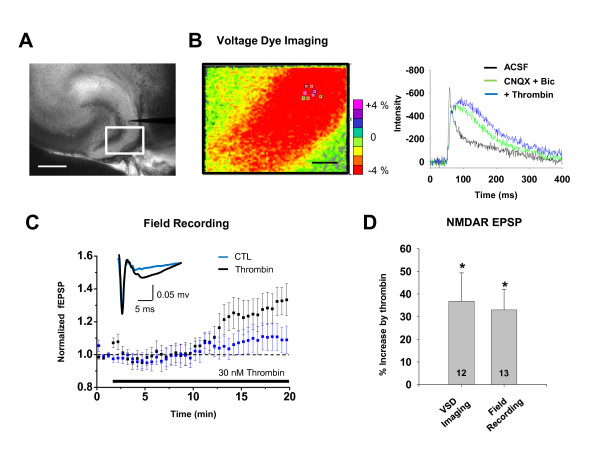
**Voltage-dye recordings demonstrate that PAR1 activation causes an increase in amplitude of NMDA receptor-mediated EPSP in dentate of hippocampus**. **A**) Bright field image of mouse dentate hippocampus, loaded with the voltage-dye, di-4-ANEPPS. The boxed region is imaged during electrical stimulation with a stimulating electrode, as shown. Scale bar is 300 μm. **B) **The left panel is voltage-dye image during a synaptic response in the presence of 10 μM bicuculline, and 10 μM CNQX. Changes in intensity correspond to degree of voltage changes and are represented in pseudo-color. The scale bar is 50 μm. The traces (*right panel*) show the time course of intensity changes averaged from pixels in a selected region of interest over the cell body layer. The time courses for each condition are overlaid and plotted; all waveforms are corrected using a baseline slope from unstimulated records to account for time-dependent changes in voltage dye fluorescence. The experiment was performed from four different mice, respectively. **C) **The time course of normalized field EPSP amplitude in the presence of 2 μM CNQX. Black trace represents the thrombin treated slice. Blue trace represents control slice. Slices were from six mice (control) and eight mice (thrombin). **D) **Similar experiments are repeated and the results are summarized in this bar graph. Bar graphs show % increase over control of NMDA EPSP by voltage dye imaging or field recording. The average % increase is expressed as mean ± SEM (* p < 0.05).

To assess the effects of PAR1 activation on synaptic transmission, we measured EPSPs in mouse dentate granule cells in response to perforant path stimulation. We measured the EPSP waveform using voltage-sensitive dye (di-4-ANEPPS) imaging of hippocampal slices, which circumvents potential artifacts associated with dialysis of the intracellular solution that might confound PAR1 signalling within dentate granule cells. The perforant path was electrically stimulated using a monopolar platinum electrode (see *Methods*), and dye fluorescence intensity change was recorded using a high speed camera with a frame transfer rate of 1 kHz (Figure 5A~B). A region of interest in the middle of the dentate molecular layer was selected at which to record the EPSP waveform, and the time course of intensity changes of several pixels recorded as a function of time (Figure 5B). The resulting waveform had the hallmark features of EPSP recorded with an intracellular pipette, but without the disturbance on intracellular signaling pathways associated with impalement and potential dialysis of the neuron.

We initially measured non-NMDA receptor-mediated EPSPs in the presence of bicuculline to block GABA_A _receptors. We subsequently supplemented the recording solution with 10 μM CNQX and reduced the extracellular Mg^2+ ^to 5 μM to isolate the NMDA receptor component of the evoked EPSPs. Following recording of EPSPs during a control period, we applied 30 nM thrombin to activate PAR1 to slices while recording the pharmacologically-isolated NMDA receptor-mediated component of the evoked EPSP. We found that PAR1 activation by thrombin increased the amplitude of the NMDA receptor-mediated EPSP (Figure 5B, D; 137 ± 14% of control; p < 0.009, paired t-test; n = 12), whereas the amplitude of the AMPA receptor-mediated EPSP was only minimally altered by thrombin (data not shown; 113 ± 5% of control, p < 0.03, paired t-test; n = 9).

To confirm the result we obtained imaging voltage-sensitive dye imaging, we performed field potential recordings in the dentate molecular layer of hippocampal slices in response to stimulation of the perforant path. To isolate NMDA receptor-mediated field EPSP, 2 μM CNQX was added and concentration of Mg^2+ ^was reduced to 0.1 mM. PAR1 activation by 30 nM thrombin significantly increased the amplitude of field EPSPs compared to slices recorded in ACSF alone (Figure 5C~D; p < 0.05, unpaired t-test; n = 12-13). This result is consistent with data from voltage-dye recordings, and confirms that PAR1 activation can enhance the NMDA component of excitatory transmission at the perforant path-granule cell synapse.

## Discussion

The most important finding of this study is that PAR1 activation induces both an increase in intracellular Ca^2+ ^and depolarization accompanied by spike firing in a subset of dentate granule neurons. In addition, PAR1 activation enhances the NMDA receptor component of the perforant path-granule cell EPSP in the dentate gyrus. The sum of these actions will be to enhance both the excitability of the dentate gyrus and the excitatory drive reaching the hippocampus. These data provide the first description of the functional effects of PAR1 activation on the dentate granule neurons.

Previous immunohistochemical studies have shown that PAR1 protein is expressed primarily in human astrocytes of white and gray matter in the cortex, hippocampus, caudate, putamen, and cerebellum [8]. By contrast, larger pyramidal neurons of the hippocampus and cortex show only modest PAR1 immunoreactivity in human brain [8]. Our immunocytochemical data indicate that PAR1 is also expressed in the dentate granule neurons, consistent with in situ hybridization data suggesting PAR1 expression in dentate gyrus [11,12]. Moreover, the immunohistochemical data are consistent with electrophysiological recordings from hippocampal slices, in which a subset of granule neurons show functional responses to PAR1 activation.

### PAR1 activation and Ca2+ increase

The PAR1 receptor is functionally linked to multiple G-proteins, including Gα_q/11_. PAR1 activation leads to a cleavage of PIP2 by phospholipase (PLC) to generate 1, 2-diacylglycerol (DAG) and inositol 1, 4, 5 triphosphate (IP3). Activation of IP3 receptors increases intracellular Ca^2+^. Thus, the observation that a subset of NMDA-responsive acutely dissociated cells shows an increase in intracellular Ca^2+ ^in response to PAR1 activation suggests that dentate neurons express PAR1 that is functionally coupled to Gα_q/11_. These data are consistent with previous study of cultured neurons from the hippocampal formation, that included the dentate gyrus [28]. In addition, data obtained from acutely dissociated cells studied here strongly suggest that PAR1 is functionally expressed in intact tissue, since the acute dissociation protocol eliminates potential confounds associated with culture conditions that could alter PAR1 expression. However, neuronal data from slice recordings showing strong depolarization following PAR1 activation raises the possibility that some of the increase in intracellular Ca^2+ ^we observe may have been supplemented by transmembrane flux through voltage gated Ca^2+ ^channels that could be activated by neuronal depolarization. There is another possible mechanism of Ca^2+ ^increase by PAR1 activation. It has been reported that Ca^2+ ^influx from extracellular region elicits Ca^2+ ^induced Ca^2+ ^release (CICR) through ryanodine receptors in the dentate gyrus [29]. Therefore, Ca^2+ ^induced Ca^2+ ^release may contribute to PAR1 induced Ca^2+ ^increase in the dentate gyrus.

### PAR1 activation and neuronal excitability

The depolarization of dentate granule cells by PAR1 activation could reflect the sum of multiple mechanisms. It has been suggested that activation of the H1 receptor, which is linked to Gαq signalling pathways, induces neuronal depolarization by blocking background K^+ ^channel [30-32]. PAR1 could engage this same pathway in a subset of granule cells to lead to modification of K^+ ^channel activity and subsequent depolarization. In addition, PAR1-mediated increases in intraneuronal Ca^2+ ^could enhance the activation of a non-selective cation current, such as those mediated by TRP channels [33]. We and others have also described a mechanism by which PAR1 activation in astrocytes triggers release of ~1 μM glutamate into the extracellular space [9,17,34]. This astrocyte-derived glutamate release has also been shown to activate NMDA receptors on neurons, which can lead to neuronal depolarization. Thus, multiple possible mechanisms exist by which PAR1-mediated increases in granule cell intracellular Ca^2+ ^could depolarize these neurons.

PAR1 activation increases the synaptically-evoked NMDA receptor-mediated component of the EPSP produced by perforant path stimulation. The PAR1-triggered enhancement in the evoked EPSP was blocked by APV and higher Mg^2+ ^concentration (data not shown), suggesting that depolarization-induced relief of Mg^2+ ^blockade may be required for thrombin-induced potentiation of NMDAR. Several previous studies have described a new mechanism by which astrocytic PAR1 activation can evoke Ca^2+^-dependent glutamate release from astrocyte that subsequently controls synaptic NMDA receptor function in neurons [9,17]. Furthermore, D-seine, an NMDAR glycine site agonist, released from astrocyte in a Ca^2+ ^dependent manner can also regulate the function of NMDA receptor [35]. The depolarization of dentate granule neurons will reduce Mg^2+ ^blockade of NMDA receptor, resulting in an apparent enhancement of the synaptic NMDA receptor response in the presence of Mg^2+^. Our data are consistent with the idea that this mechanism is operational in the dentate gyrus.

In addition PAR1 activation could also lead to the generation of lysophosphatidic acid and arachidonic acid, a mechanism that previously has been described in platelets and endothelial cells [36]. Both of these lipid signaling molecules are highly mobile and capable of mediating intercellular signaling. Although it is not yet known whether PAR1 activation in dentate gyrus can trigger formation of these lipids, release of lysophosphatidic acid or arachidonic acid could potentially play a role in the effects described here. Whereas arachidonic acid can directly potentiate neuronal NMDA receptor function [37], lysophosphatidic acid has been suggested to enhance NMDA receptor function through depolarization-induced relief of Mg^2+ ^block [38]. Further work is needed to determine the relative contribution of these (and other) mechanism(s) to PAR1-induced depolarization of dentate granule cells.

### Function of PAR1 in physiological and pathological conditions

The physiological role of PAR1 receptors in normal synaptic transmission has not been extensively investigated. Interestingly, tissue plasminogen activator (tPA), which is known to convert plasminogen to plasmin, can influence long-term potentiation, an NMDA receptor-dependent cellular model of learning and memory [39]. Several lines of evidence indicate that plasmin can regulate the function of NMDA receptors through PAR1 activation [34,40]. Similarly, PAR1 -/- mice show deficiencies in emotional learning, an NMDA receptor-dependent process [41]. Taken together, these results suggest that tPA-activated plasmin could be an endogenous ligand for PAR1 receptor [20] that leads to PAR1-mediated tuning of NMDA receptor function in a manner relevant for synaptic plasticity and behavior. By potentiating synaptic NMDA receptor, we predict that PAR1 activation would decrease threshold for stimuli needed to trigger changes in synaptic strength. Consistent with this idea, multiple studies have shown that PAR1 activation can influence the threshold for LTP [42,43]. In addition, sufficient thrombin can enter brain tissue in pathological conditions such as ischemia or hemorrhage and activate PAR1 receptor, which in multiple animal models has been shown to enhance neuronal damage [44-54]. During ischemia, the harmful actions of PAR1 require NMDA receptor function [55]. Our data are consistent with the idea that PAR1 activation in the dentate gyrus can enhance neuronal excitability, which may promote NMDA-receptor mediated excitotoxicity in neurons [43,45,56].

## Methods

### Immunohistochemistry

Wild type C57Bl/6 mice were deeply anesthetized by 2% avertin (20 μl/g) and transcardially perfused with 4% paraformaldehyde. All procedures involving the use of animals were reviewed and approved by the Emory University IACUC. The brain was isolated, and cut at 30 μm with a cryostat. Sections were blocked in 0.1 M PBS containing 0.3% triton X-100 (Sigma) and 2% serum from species of the secondary antibody for 1 hr. Thrombin receptor goat polyclonal antibody against mouse PAR1 N-terminal (S-19; Santa Cruz; catalog # sc-8204) was applied at 1:20 dilution (S-19) and incubated overnight at 4°C. After overnight incubation, the sections were washed three times in phosphate-buffered saline (PBS) and then incubated in secondary antibody (Alexa 555 donkey anti-'goat IgG; Invitrogen; 1:400) for 2 hr. After three rinses in PBS, the sections were mounted on slide glass. Images were acquired on an Olympus Fluoview FV1000 confocal microscope and analyzed using Image J software.

Adult Sprague-Dawley rats were given a lethal dose of pentobarbital (150 mg/kg) and subsequently transcardially perfused with cold 3% paraformaldehyde (4°C). The brain was subsequently isolated, post-fixed in 3% paraformaldehyde for 24 hrs, cryoprotected in 30% sucrose and 0.1 M phosphate, frozen on dry ice, and cut at 40 μm on a sliding microtome. The sections were washed 6 times (10 min each) in Tris-buffered saline (TBS), and incubated in 3% H_2_O_2 _to block endogenous peroxidases. The sections were washed 3 times (10 min each) in TBS plus 0.1% Triton X-100, followed by incubation with the monoclonal WEDE15 PAR1 antibody (5 μg/ml, Immunotech-Coulter; epitope residues 51-64 in exon2, KYEPFWEDEEKNES) in 10% horse serum in TBS overnight at 4°C. The sections were washed 3 times (10 min) in TBS and incubated for 1 hr in biotin-conjugated rat anti-mouse secondary antibody (1:200) in 10% horse serum in TBS plus 0.1% Triton X-100. The sections were washed 3 times (10 min) in TBS and the avidin-biotin complex method was used to detect antigen signal. 3,3'-diamino benzidine tetrachloride was used to visualize the final product. Immunostained sections were mounted on slides and visualized using bright field microscopy; data were obtained from three independent experiments, and showed similar staining patterns. No immunoreactivity could be detected when either the primary or secondary antibody was omitted from the protocol (data not shown). Inclusion of a peptide matching the epitope blocked staining in dentate gyrus (data now shown).

### Primary astrocyte culture

Cultured astrocytes were prepared from P0~P3 postnatal mice. The cerebral cortex was dissected free of adherent meninges, minced and dissociated into single cell suspension by trituration. Dissociated cells were plated onto 12 mm glass coverslips coated with 0.1 mg/ml poly D-lysine. Cells were grown in DMEM supplemented with 25 mM glucose, 10% heat-inactivated horse serum, 10% heat-inactivated fetal bovine serum, 2 mM glutamine, and 1000 units ml^-1 ^penicillin-streptomycin. Cultures were maintained at 37°C in humidified 5% CO_2_-containing atmosphere. Astrocyte cultures prepared in this way were confirmed by GFAP staining using anti-GFAP antibody (Millipore; catalog # AB5541; 1:1000)

### Western blotting

Adult rat and mouse brain regions were dissected, homogenized in ice cold RIPA buffer (phosphate buffered saline, 1% Igepal CA-360, 0.5% Na-deoxycholate, 0.1% SDS) containing the protease inhibitor 1 mM PMSF. The membranes were stored at 20°C. Whole cell lysate was incubated with 2% SDS, 62.5 mM Tris, 10% glycerol, 5% β-mercaptoethanol, and 0.05% bromophenol blue for 5 minutes at 100°C. 40 μg of protein for each sample was loaded and separated on 10% polyacrylamide gels, then transferred to PVDF membranes. Blots were blocked with TBS containing 1% Tween-20 and 5% skim milk for 30 min at RT, and incubated with a goat polyclonal antibody against thrombin receptor (S-19; Santa Cruz; 1:300) overnight at 4°C. After washing with TBS plus Tween, blots were incubated with HRP-conjugated anti-goat secondary antibody (Santa Cruz; 1:3000), followed by washing and the detection of immunoreactivity with enhanced chemiluminescence (ECL; Amersham). The same blots were re-probed with a rabbit monoclonal antibody against α-actin (Sigma; catalog # A2066; 1:2000) to confirm equal loading.

### Ca^2+ ^Imaging

The dentate gyrus was dissected from 400 μm horizontal rat brain slices, incubated with 1 mg/ml trypsin for 20 min, and then mechanically dissociated using vibration with fire polished glass pipettes. Dissociated cells are plated on glass coverslips, and loaded with 5 μM Fluo3-AM for 30 min for Ca2+ imaging. External solution contained (in mM) 150 NaCl, 10 HEPES, 3 KCl, 2 CaCl_2_, 1 MgCl_2_, 22 sucrose, 10 glucose; pH adjusted to 7.4 and osmolarity to 325 mOsm (23°C). 30 μM TFLLR or 100 μM NMDA were applied to the cells Fluo3 was excited by a 100 W mercury lamp and the timing was controlled by a high speed shutter (Uniblitz). Images were acquired and analyzed by custom software.

### Whole-cell patch clamp recording of dentate granule cells

Young mice (C57/B16, age P15-20) were deeply anaesthetized with isoflurane until cessation of breathing and subsequently decapitated. The brain was rapidly removed and submerged in an ice-cold oxygenated artificial cerebrospinal fluid (ACSF) composed of (in mM) 130 NaCl, 24 NaHCO_3_, 3.5 KCl, 1.25 NaH_2_PO_4_, 1 CaCl_2_, 3 MgCl_2_, 10 glucose at pH 7.4, and was bubbled with 5% CO_2_/95% O_2_. Transverse slices (300 μm) were prepared with a Leica vibratome, and incubated in a chamber with oxygenated ACSF at room temperature for 1 hr before use. The internal solution was comprised of (mM) 140 K-MeSO_4_, 10 HEPES, 7 NaCl, 4 Mg-ATP, and 0.3 Na-GTP. The recording ACSF solution was composed of (in mM) 130 NaCl, 24 NaHCO_3_, 3.5 KCl, 1.25 NaH_2_PO_4_, 1.5 CaCl_2_, 1.5 MgCl_2_, and 10 glucose at pH 7.4 and was bubbled with 5% CO_2_/95% O_2 _(23°C). Visually guided whole-cell patch recordings were obtained from dentate granule neurons in current clamp configuration using an Axopatch 200A (Axon instruments, Union City, CA, USA) and a patch pipette of 5~7 MΩ resistance. Electrophysiological properties were monitored before and at the end of the experiments. Series and input resistances were monitored throughout the experiment using a -5 mV pulse. Recordings were considered stable when the series and input resistances, resting membrane potential and stimulus artifact duration did not change > 20%.

### Voltage Dye Imaging

400 μm thick transverse hippocampal slices of mouse brain were loaded with the voltage dye, di-4-ANEPPS (D-1199, Molecular Probes Inc.) at 3 mg/ml for 10-30 min. The voltage sensitive dye (VSD) was dissolved into a 2:1 mixture of ethanol and 10% Cremophor EL (Sigma), a castor oil derivative, which was used as a dye stock solution (3.3 mg of VSD/ml mixture). The dye stock solution was mixed with a 1:1 mixture of ACSF and fetal bovine serum (Sigma) to a final VSD concentration of 0.2 mM and was used as staining solution. Each slice was stained with 100 ml of the staining solution by gently squirting the solution into the plexiglass ring, following incubation in a humidified chamber for 25 min. The slices were rinsed with ACSF by dipping it together with the plexiglass ring [57]. Images (resolution: 60 × 90 pixels) were acquired at 100 Hz (10 ms sample interval) for 65 seconds (23°C), using MiCam camera system (BrainVision, Japan). This high speed camera system converted the changes in intensity to different colors. Di-4-ANEPPS decreases its fluorescence intensity when the membrane depolarizes. A 20× water objective (N.A. = 0.95, Olympus) was used to acquire the images. The dye was excited at 510 nm and emitted light that was detected at 590 nm.

#### Field Recording

400 μm transverse hippocampal slices were prepared from 21-28 day old mice, as described above. Slices (400 μm thick) were placed in a submerged chamber superfused with oxygenated ACSF (see above). Field potentials were recorded with a micropipette (5-10 MOhm) filled with HEPES-buffered saline positioned in the dentate gyrus molecular layer. Field EPSPs were evoked at room temperature (25°C) by perforant path stimulation (0.1 ms, 10-100 μA) using a monopolar stimulating electrode. The NMDA component was isolated by recording in 0.1 mM Mg^2+ ^and 10 μM CNQX. NMDA-component field EPSPs were digitized at 10 kHz, and the field EPSP amplitude quantified.

## Competing interests

The authors declare that they have no competing interests.

## Authors' contributions

KSH carried out immunohistochemistry and wrote the manuscript. GM performed whole cell patch from dentate granule neuron. CEH carried out voltage sensitive dye imaging. JL performed the immunoblotting and immunostaining in cultured astrocyte. CEJ carried out the immunoblotting. CJL performed Ca2+ imaging and field recording. SFT designed the most of experiments and wrote the manuscript, and coordinate entire project. All authors read and approved the final manuscript.
